# Study and Experiment on Non-Contact Voltage Sensor Suitable for Three-Phase Transmission Line

**DOI:** 10.3390/s16010040

**Published:** 2015-12-30

**Authors:** Qiang Zhou, Wei He, Dongping Xiao, Songnong Li, Kongjun Zhou

**Affiliations:** 1 State Key Laboratory of Power Transmission Equipment & System Security and New Technology, Chongqing University, Chongqing 400044, China; weihe2016@126.com (W.H.); dongpingxiao2016@sina.com (D.X.); 2 State Grid Chongqing Electric Power CO. Electric Power Research Institute, Chongqing 400015, China; songnongli2016@126.com (S.L.); kongjunzhou2016@sina.com (K.Z.)

**Keywords:** voltage sensor, non-contact measurement, ansoft maxwell, electric field intensity, three phase transmission lines

## Abstract

A voltage transformer, as voltage signal detection equipment, plays an important role in a power system. Presently, more and more electric power systems are adopting potential transformer and capacitance voltage transformers. Transformers are often large in volume and heavyweight, their insulation design is difficult, and an iron core or multi-grade capacitance voltage division structure is generally adopted. As a result, the detection accuracy of transformer is reduced, a huge phase difference exists between detection signal and voltage signal to be measured, and the detection signal cannot accurately and timely reflect the change of conductor voltage signal to be measured. By aiming at the current problems of electric transformation, based on electrostatic induction principle, this paper designed a non-contact voltage sensor and gained detection signal of the sensor through electrostatic coupling for the electric field generated by electric charges of the conductor to be measured. The insulation structure design of the sensor is simple and its volume is small; phase difference of sensor measurement is effectively reduced through optimization design of the electrode; and voltage division ratio and measurement accuracy are increased. The voltage sensor was tested on the experimental platform of simulating three-phase transmission line. According to the result, the designed non-contact voltage sensor can realize accurate and real-time measurement for the conductor voltage. It can be applied to online monitoring for the voltage of three-phase transmission line or three-phase distribution network line, which is in accordance with the development direction of the smart grid.

## 1. Introduction

The voltage transformer is an important part of an electric power system and the detection performance of the transformer can directly influence the reliability of an electric power system voltage measurement and relay protection device motion as well as the accuracy of electric power measurement. Presently, voltage transformers extensively applied to electric power system mainly include potential transformer and capacitance voltage transformer [1,2,3,4,5].

**Figure 1:**
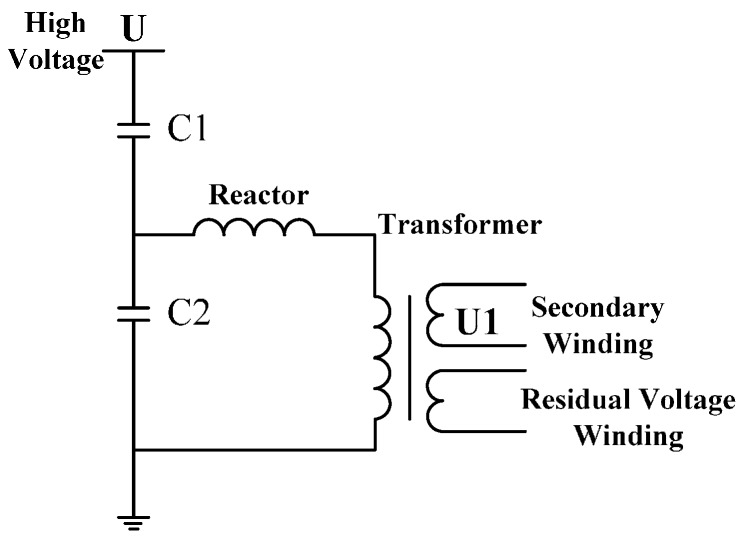
Structure diagram of CVT.

Structure diagram of Capacitance Voltage Transformer (CVT) is shown in Figure 1. The high voltage terminal is directly connected to electrified body and the other terminal is connected to the ground; C1 and C2 are adopted for capacitance voltage division, and voltage signal after voltage division will be transformed into output signal of transformer through potential transformer [6,7,8,9].

**Figure 2:**
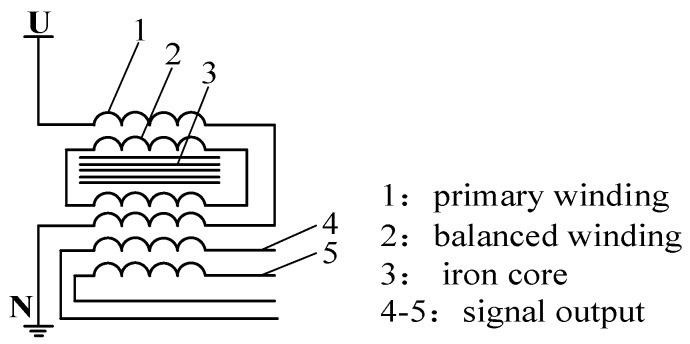
Potential transformer.

Potential transformer is shown in Figure 2, and the high voltage terminal is connected to electrified body.

According to analysis the Figure 1 and Figure 2, the traditional voltage transformers shown in Figure 1 and Figure 2, the high voltage terminal of currently used voltage transformer has direct electrical connection with conductor to be measured, and the other terminal of the transformer is connected to the ground. Therefore, insulation design of the transformer is difficult, and, meanwhile, it involves a large volume and heavyweight; due to the iron core design and multi-grade capacitance voltage division of the transformer, serious time delays occur during the detection signal and voltage signal to be measured in phase position. As a result, detection accuracy of the transformer is reduced and its application range is restricted. Moreover, three-phase voltage transformer is seldom adopted for the voltage grade above 35 kV in electric power systems. Therefore, traditional voltage transformers can no longer satisfy the development of the electric power system [10,11,12].

**Figure 3:**
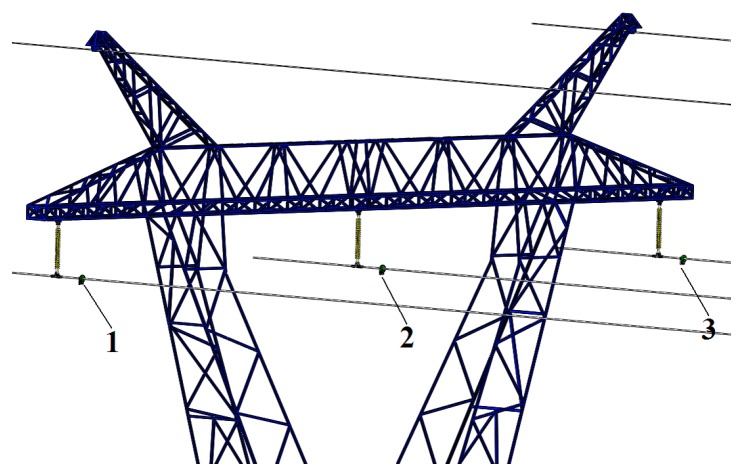
Application of voltage sensor in transmission line.

In consideration of the above-mentioned problems, this paper designed a non-contact voltage sensor on the basis of electric field coupling principle, as shown in Figure 3. In Figure 1, Figure 2 and Figure 3 are non-contact voltage sensors installed on the three-phase transmission line. Positive and negative induction electrodes are designed on the sensor, and the electric field produced by electric charges on the transmission line will generate induced charges on the electrode of voltage sensor by way of electrostatic induction. The electric charges will distribute on the electrode to form electric potential, and the potential signal of positive and negative induction electrodes is output signal of the non-contact voltage sensor.

Compared with traditional voltage transformer, non-contact voltage sensor has no electrical connection with electrified body. Therefore, the insulation design is simple and the cost is low; the induction electrode of non-contact voltage sensor is optimized through simulation calculation. Miniature design of the sensor is realized, the voltage division ratio is increased, and phase error of sensor detection signal is reduced. An experimental platform that consisted of a 50 kV simulated three-phase transmission line was established in the laboratory to test the non-contact voltage sensor designed in this paper. According to the result, the designed voltage sensor can realize real-time detection for the voltage of three-phase transmission line and accurately measure the voltage and phase position.

**Figure 4:**
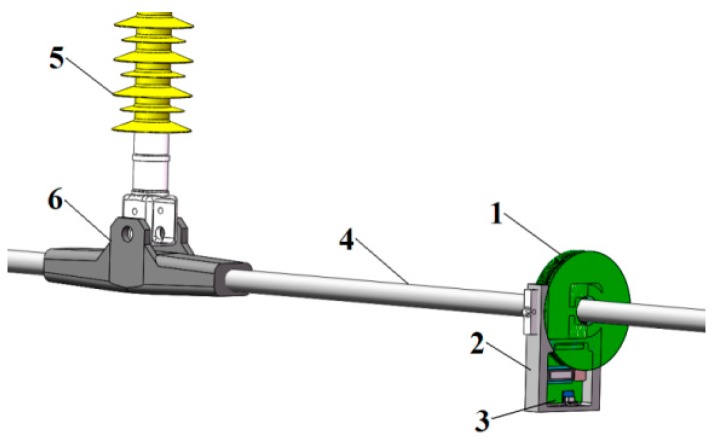
Non-contact open-type voltage sensor.

Non-contact voltage sensor used at the laboratory test stage adopts closed annular electrode, and it is mainly applied to detection performance and insulation performance test of the sensor. In the follow-up study, open-type annular electrode presented in Figure 4 will be designed. The sensor can be conveniently installed on the line, while the signal detection and processing circuit and wireless transmission circuit will be designed. In this way, the detection signal can be transferred to the control terminal in time. In Figure 4, 1 refers to non-contact voltage sensor; 2 is the insulation fixture—rubber with good insulation performance and toughness is used in the center fixture so that when the fixture is compressed, the centers of the line and sensor electrode can be maintained in the coaxial position, owing to the existence of non-conducting rubber; 3 indicates the signal processing circuit, wireless transmission module and battery; 4 is power transmission line; 5 is the insulator; and 6 denotes the connection fitting.

## 2. Detection Principle

The surface of any metal electrified conductor has electric charges that will generate electric field. In electric engineering, the power frequency electric field of 50 Hz can be treated as quasi static electric field. At this time, when metal electrode is introduced into this electric field, induced charges will be produced on the metal electrode, and the quantity of electric charges will change with the variation of electric field intensity. Distribution of electric charges on the metal surface will form electric potential of the metal conductor. When there is potential to ground in this environment, potential difference between these two is the induced voltage of metal electrode.

**Figure 5:**
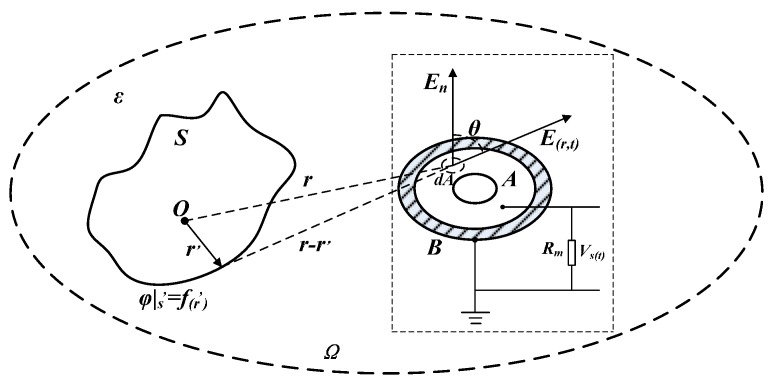
Principle of sensor measurement.

A non-contact voltage sensor was designed according to the above electrostatic induction principle. Different from the traditional sensor that directly measures the voltage of electrified conductor, this sensor couples electric field produced by electric charges of the conductor to be measured into voltage sensor, so as to form detection signal. Figure 5 is the schematic diagram for the principle of sensor measurement.

In Figure 5, S indicates the external surface boundary of electrified conductor in any shape; Ω means the computational domain of the whole electric field; ε refers to the electric medium constant in the area; *r* signifies the position vector of the calculation site; r’ denotes the position vector of original point; *E*(*r*) is the electric field intensity of any point in the computational domain; *F*(*r’*) refers to the potential distribution on the external surface of electrified conductor; *φ_0_*(*t*) means the electric potential of electrified conductor; A indicates the external surface of metal electrode of the voltage sensor; B is the sensor support (connected to the ground); and *R_m_* denotes the sampling resistance of voltage sensor.

No free charges exist in the computational domain, *i.e.*, *ρ*(*r’*) = 0. The computational domain meets progressive boundary conditions, and any point in the computational domain meets Poisson equation of Equation (1):

(1) $$\nabla^{2}\varphi_{\left(r\right)}=-\frac{\rho_{\left(r\right)}}{\varepsilon }$$

Green function is introduced and the following result is gained by solving Equation (1) through the first boundary condition *φ|s’* = *f*(*r’*):

(2) $$\varphi_{0}=\frac{1}{\varepsilon F(r)}E(r)$$

In Equation (2), *F*(*r*) is decided by the distance of field point from the conductor to be measured. According to this equation, in the computational domain Ω, the electric field intensity of any point is in direct proportion to electric potential of the conductor to be measured, and they have a linear relation.

Suppose that the electric potential change of electrified conductor with time is *φ_0_*(*t*) and the frequency is 50 Hz; at this time, the electric field produced by electrified body can be treated as quasi static field. Under the effect of this electric field, induced charge q will appear on the surface of metal electrode A due to electrostatic induction principle. Closed Gaussian surface is created on the electrode surface with the distance of r from the electrified conductor, and the differential element dA is taken [13,14,15]. The electric field intensity component at normal direction is *E_n_* and the angular separation with *E*(*r,t*) is *θ*. The following equation can be gained according to Gauss theorem:

(3) $$\frac{dq}{dt}=\oint_{A}\varepsilon \frac{dE(r,t)}{dt}dA$$

According to Equation (3), the electric field intensity produced by electric charges on the energized conductor changes with the time *t*, and the electric field intensity will cause corresponding changes to the quantity of electric charges on induction electrode A. The induced charges will distribute on electrode A to form electric potential *φ_s_*(*t*). Sampling resistance *R_m_* is used to connect induction electrode A and support B, and the voltage *V_s_*(*t*) at both ends of the sampling resistance is the detecting voltage:

(4) $$V_{s}(t)=R_{m}\oint_{A}\varepsilon \frac{dE(r,t)}{dt}dA=\frac{\varepsilon_{0}Ar^{\prime }}{r^{2}}R_{m}\frac{d}{dt}\varphi_{0}(t)$$

By organizing Equation (4), the following equation can be gained:

(5) $$\varphi_{0}(t)=\frac{r^{2}}{\varepsilon AR_{m}r^{\prime }}\int V_{s}(t)dt$$

When the electrified conductor is cylindrical transmission line conductor, *r*’ is the section radius of the cylinder. At this time:

(6) $$K=\frac{r^{2}}{\varepsilon AR_{m}r^{\prime }}$$

In Equation (6), *K* is a constant, so:

(7) $$\varphi_{0}(t)=K\int V_{s}(t)dt$$

According to Equation (7), the voltage value of electrified conductor can be calculated by conducting integral operation for the voltage signal of sampling resistance and multiplying the result by *K*; these two have a linear relation. According to the above discussion, a non-contact method can be found to realize non-contact measurement for the voltage of power frequency high voltage electrified body.

## 3. Design of Non-Contact Voltage Sensor

**Figure 6:**
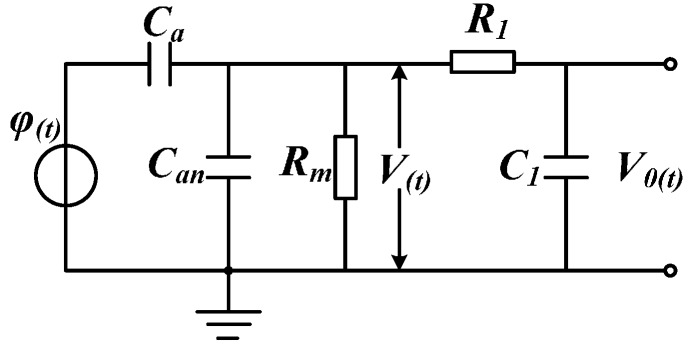
Equivalent circuit model.

Figure 6 presents the lumped parameter equivalent circuit model of electric field coupling sensor based on electrostatic induction. *φ*(*t*) refers to the equivalent voltage of the conductor to be measured; *C_a_* means the equivalent mutual capacitance between the sensor electrode and conductor to be measured; *C_an_* indicates the ground equivalent stray capacitance of sensor electrode; *R_m_* signifies the detecting resistance; and *R*_1_ and *C*_1_ constitute integral circuit and the output signal of integral circuit is *V*_0_(*t*), respectively.

Through Laplace transformation, transfer Equation (8) of sensor and transfer Equation (9) of passive integral circuit can be gained:

(8) $$H_{M}(s)=\frac{C_{a}R_{m}s}{1+(C_{a}+C_{an})R_{m}s}$$

(9) $$H_{N}(s)=\frac{1}{1+C_{1}R_{1}s}$$

Suppose that ω_h_ and ω_l_ are upper limit and lower limit of sensor measurement bandwidth; the following equations can be gained:

(10) $$\omega_{H}=\frac{1}{(C_{a}+C_{an})R_{m}}$$

(11) $$\omega_{L}=\frac{1}{C_{1}R_{1}}$$

**Figure 7:**
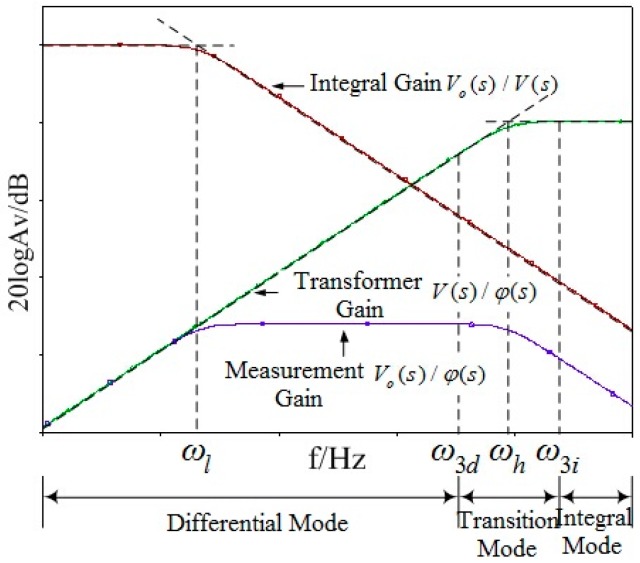
Amplitude-frequency response curve.

The characteristic amplitude-frequency response curve of the sensor, integral circuit and the entire measurement system can be obtained through Equations (8) and (9), as shown in Figure 7.

As shown in Equation (12), when (*C_a_* + *C_an_*)*R_m_* << 1, the sensor works under differential mode:

(12) $$H_{M}(s)=\frac{V(s)}{\varphi (s)}=C_{a}R_{m}s$$

At this time, in order to meet the measurement requirements, integral circuit should be added, but integral circuit will cause troubles to signal-to-noise ratio design of sensor. Meanwhile, due to problems of component parameters in integral circuit, detecting waveform distortion and inaccurate measurement problems will be caused. Therefore, sensor should work under self-integration mode. In other words, Equation (13) is gained when (*C_a_* + *C_an_*)*R_m_* >> 1. At this time, the required detection signal of sensor can be obtained without integral circuit.

(13) $$H_{M}(s)=\frac{V(s)}{\varphi (s)}=\frac{C_{a}}{C_{a}+C_{an}}$$

In order to make the sensor work under self-integration mode at the power frequency of 50 Hz, the angular frequency should be *ω_H_* < 2π × 50. Therefore, (*C_a_* + *C_an_*)*R_m_* must meet the following condition:

(14) $$(C_{a}+C_{an})R_{m}>3.18\times 10^{-3}$$

Non-contact measurement mode is adopted, so the distance between the sensor and conductor to be measured cannot be short. Thereby, the mutual capacitance *C_a_* between the conductor to be measured and induction electrode cannot be large and the distance between the sensor and ground is long. Therefore, the ground stray capacitance *C_an_* of the sensor is not large (generally speaking, *C_a_* and *C_an_* are at the level of pF). In order to meet the condition of Equation (14), the sampling resistance *R_m_* should be at the level of GΩ, which will cause problems to equivalent internal resistance detection and impedance matching of the sensor. The small capacitance values of *C_a_* and *C_an_* also restrict the voltage division ratio of sensor. One terminal of the sampling resistance is connected to the ground, increasing the difficulty of the insulation design of the sensor. Most existing electric field coupling sensors based on electrostatic induction adopt the form of adding integral circuit, so as to gain the signal to be detected. Meanwhile, the sensor electrode often adopts plate or cylindrical design, which might restrict detection bandwidth, voltage division ratio, detection accuracy, and insulation design of the sensor.

**Figure 8:**
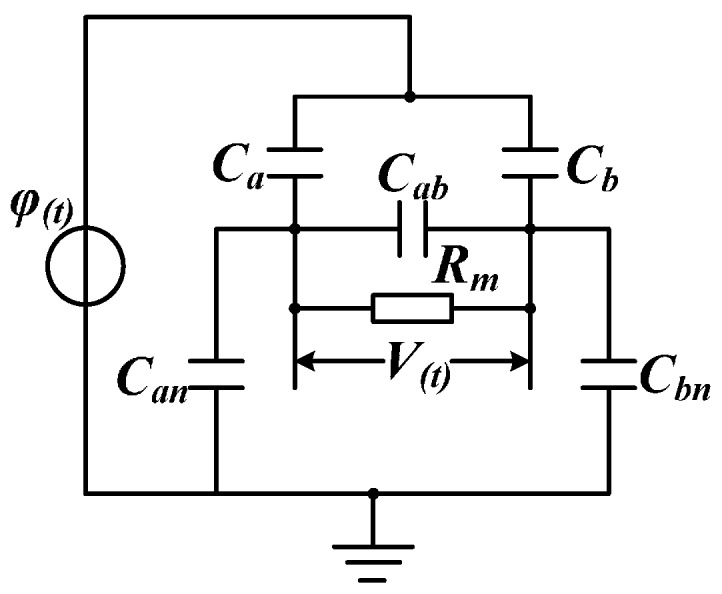
Equivalent circuit of voltage sensor.

By directing at the above problems, the equivalent circuit of non-contact voltage sensor designed in this paper is given, as shown in Figure 8. The voltage sensor of adopting differential input structure sets the difference of suspended potentials output by a pair of sensor electrodes working under self-integration mode as the output of sensor. In Figure 7, *φ*_(*t*)_ means the equivalent voltage source of the conductor to be measured; *C_a_* and *C_b_* refer to the equivalent mutual capacitance between induction electrode and conductor to be measured; *C_an_* and *C_bn_* signify the ground equivalent stray capacitance of induction electrode; C_ab_ indicates the equivalent mutual capacitance between electrodes; Mark 1 denotes the suspended potential of voltage source; Marks 2 and 3 are suspended potentials output by two electrodes; and *R_m_* refers to the detecting resistance [16].

Transfer function of the system is obtained through Laplace transformation:

(15) $$H(s)=\frac{(C_{a}C_{an}-C_{b}C_{bn})R_{m}s}{(C_{ab}R_{m}s+1)(C_{a}+C_{b}+C_{an}+C_{bn})+(C_{a}+C_{an})(C_{b}+C_{bn})R_{m}s}$$

According to Figure 8, the non-contact voltage sensor designed in this paper adopts two induction electrodes and inputs suspended potentials of the two electrodes into the differential amplifier of signal processing circuit as detection signals. In this method, no terminal of the sensor is connected to the ground, greatly reducing the insulation design difficulty of the sensor. The signal processing method of differential type can effectively eliminate the interference of common-mode signal and increase the measurement accuracy of sensor.

By comparing Equation (8) with Equation (15), after design structure of this paper is introduced, transfer function of the sensor will not be changed. Only mutual capacitance *C_ab_* between electrodes is introduced in the electrode point. Compared with the mutual capacitance *C_a_* between the induction electrode and conductor to be measured that cannot be large capacitance, the distance between induction electrodes can be designed freely and the inter-electrode capacitance *C_ab_* can be adjusted freely. Therefore, such circuit structure can meet the requirement of Equation (14), and the sensor is able to work under self-integration mode.

The amplitude-frequency characteristic function of sensor designed in this paper can be gained according to Equation (15):

(16) $$\left|H(\omega )\right|=\frac{\frac{C_{a}C_{bn}+C_{b}C_{an}}{C_{a}+C_{b}+C_{an}+C_{bn}}R_{m}}{\sqrt{R_{m}^{2}{\left[\frac{\left(C_{a}+C_{an})(C_{b}+C_{bn}\right)}{C_{a}+C_{b}+C_{an}+C_{bn}}+C_{ab}\right]}^{2}+\frac{1}{\omega^{2}}}}$$

Its phase-frequency characteristic function is:

(17) $$∠H(\omega )=\arctan\frac{1}{R_{m}\omega [\frac{\left(C_{a}+C_{an})(C_{b}+C_{bn}\right)}{C_{a}+C_{an}+C_{b}+C_{bn}}+C_{ab}]}$$

According to Equation (16), the inter-electrode mutual capacitance *C_ab_* exists in the denominator, so the transformation ratio of the sensor can be increased by magnifying the mutual capacitance *C_ab_* between electrodes without changing other conditions. In Equation (17), *C_ab_* also exists in the denominator only, so the phase difference between output signals of the electrified body to be measured and voltage sensor can be reduced by increasing the inter-electrode capacitance *C_ab_*, thus measurement accuracy and response speed of the sensor can be further enhanced.

Through the above analysis, the sensor is required to work under self-integration mode at the power frequency of 50 Hz due to the following two reasons:

(1) If the sensor works under differential state, integral circuit should be designed. According to the analysis of circuit structure, the detection bandwidth upper limit of sensor is restricted by corner frequency of the sensor at this time, and detection bandwidth lower limit is restricted by corner frequency of the circuit. Therefore, bandwidth of the entire sensor detector is restricted.

(2) Capacitance and resistance in integral circuit are restricted by factors like temperature coefficient. As a result, the accuracy of simulating integral circuit can hardly reach the requirement. Meanwhile, due to stray parameters of components and parts in integral circuit, distortion might happen to the detection signal.

According to the analysis in this paper, in order to meet the above requirements, the mutual capacitance between sensors should be increased to the largest extent. This paper shows that the method of increasing the mutual capacitance between induction electrodes of the sensor is to treat suspended potential signal of sensor electrode as output signal of the sensor. The purpose of adopting differential amplifier is to restrain common-mode signal in suspended potential signal of sensor, and to amplify the differential signal at the same time.

In the future, solar panel will be adopted as power supply of signal processing circuit. The circuit board adopts the mode of DC +5 V power supply, and the differential amplifier uses the mode of +3 V and −3 V power supply. Signal processing circuit and solar panel are designed in the box, and installed in the tower of high-voltage transmission line, so as to realize voltage detection for high-voltage transmission line.

**Figure 9:**
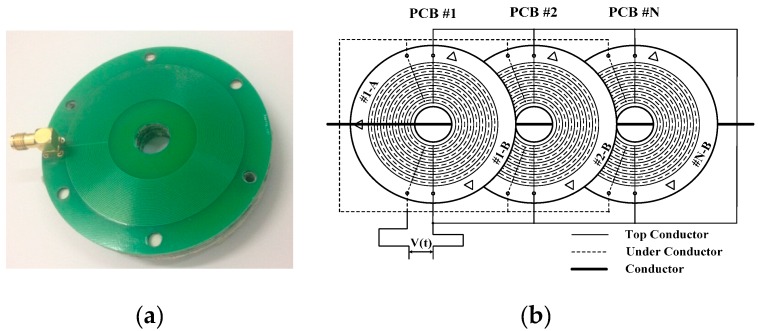
Design of non-contact voltage sensor: (**a**) physical design of the sensor; and (**b**) schematic diagram for sensor structure.

According to the above analysis, the non-contact voltage sensor designed in this paper is presented in Figure 9. Figure 9a shows the physical design of sensor. Annular copper electrodes are arranged on the front and back sides of PCB board (printed-circuit board) as induction electrodes. The sensor diameter is 70 mm and the pore diameter in the middle is 18 mm; there are 32 electrodes on the front side and the electrode spacing is 0.3 mm; there are 32 electrodes on the back side and the electrode spacing is 0.3 mm; and the detection signal is drawn forth by the screened wire. Figure 9b is the schematic diagram for sensor structure and the electric transmission line passes through the pore in the middle of the sensor. In order to enhance the mutual capacitance between electrodes and the sensor electrode ability of holding electric charges to the largest extent, multiple PCB boards are connected; all electrodes on the front side of each PCB board are connected and all electrodes on the back side of each PCB board are connected.

## 4. Simulation

In this paper, Ansoft software is used to realize optimization for the structure design of non-contact voltage sensor. Meanwhile, the influence of sensor introduction on measurement and the distortion situations around the electric field are studied when sensor is applied to voltage detection of three-phase transmission line.

The parameters of voltage sensor were shown in Table 1, and those parameters were used in simulation.

**Table 1:** Parameters of sensor.

Sensor	Number of Electrode	Width of Electrode (mil)	Spacing of Electrode (mm)	Diameter of Innermost Electrode (mm)
Board front	32	6	0.3	28
Board opposite	32	6	0.3	

The actual sensor adopts 12 PCB boards. According to the measurement results of Agilent 4294 A impedance analyzer, electrode mutual capacitance between the output ends of the PCB sensor is 950 pF, and the maximum fluctuation deviation value within the frequency range from 45 Hz to 2 MHz is 2 pF.

### 4.1. Optimization Design of Sensor Structure

**Figure 10:**
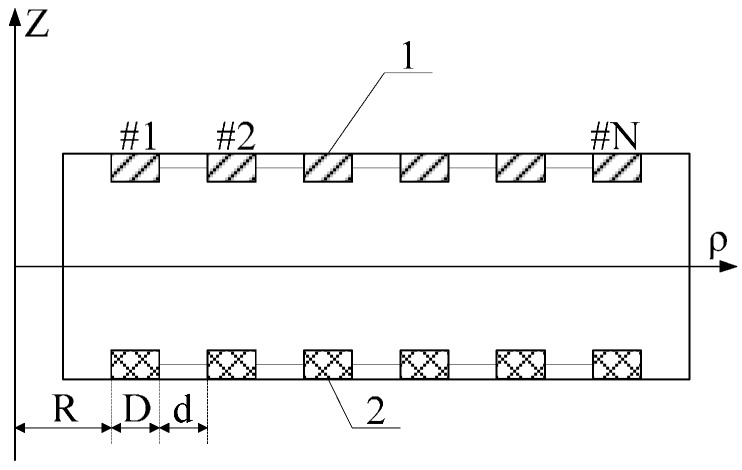
Sensor electrode structure.

**Figure 11:**
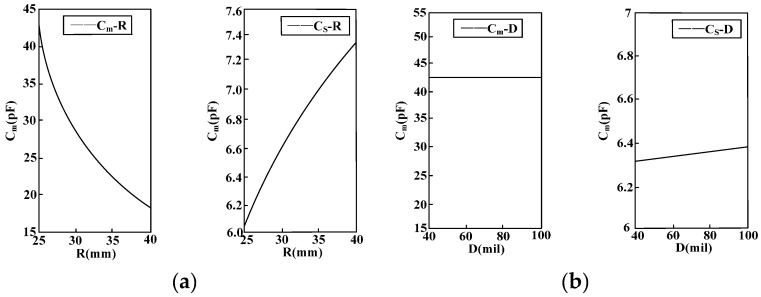

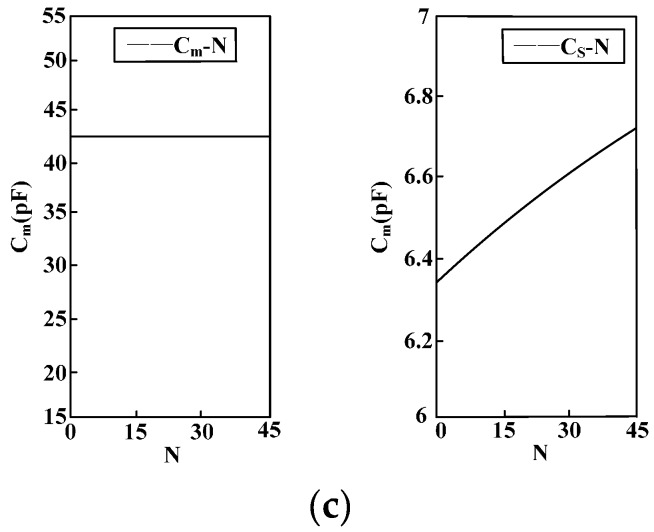
Simulation result of electrode structure optimization. (**a**) Relation curve of equivalent capacitance and electrode radius; (**b**) Relation curve of equivalent capacitance and electrode width; (**c**) Relation curve of equivalent capacitance and number of electrodes.

The simulation experiment model is established according to the electrode structure presented in Figure 10 where 1 represents the electrode on the front side of PCB board; 2 means the electrode on the back side of PCB board; R, D and d refer to the radius, width and electrode spacing of #1 electrode, respectively; and N indicates the number of electrodes. Through post-processing for the simulation result, equivalent distributed capacitances under different motivations and different electrode structures can be gained via calculation, as shown in Figure 11. Figure 11a is the relation curve of equivalent capacitance and electrode radius; Figure 11b presents the relation curve of equivalent capacitance and electrode width; and Figure 11c shows the relation between equivalent capacitance and number of electrodes.

The following conclusions can be gained through the simulation result:

(1) With the increase of the transformer electrode radius *R*, the mutual capacitance *C_ab_* between electrodes decreases and the ground stray capacitance *C_n_* of electrode rises. 

(2) With the increase of the transformer electrode width *D*, the ground stray capacitance *C_n_* of electrode rises, while the mutual capacitance *C_ab_* between electrodes remains unchanged.

(3) With the increase of the number of electrodes *N*, the ground stray capacitance *C_n_* of electrode rises, while the mutual capacitance *C_ab_* between electrodes remains unchanged.

Therefore, when the induction electrode of sensor is designed, in order to reduce the measurement error disturbance caused by the ground stray capacitance *C_n_* of sensor, the electrode radius should be reduced to the largest extent, and meanwhile the width and distance between electrodes must be decreased. On the premise of comprehensively considering factors like the size of sensor space, more electrodes should be arranged and the mutual capacitance between electrodes must be increased to the largest extent. In this way, the voltage division ratio and measurement accuracy of sensor can be increased, and phase error in measurement will be reduced.

### 4.2. Insulation Design of Sensor

#### 4.2.1. Insulation Design between Electrodes

**Figure 12:**
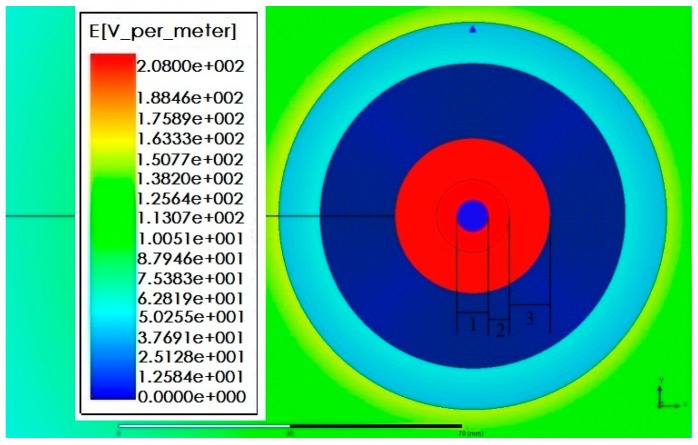
Insulation intensity of sensor electrode and simulation calculation.

In order to increase the mutual capacitance *C_ab_* between electrodes to the largest extent, measures to reduce the distance between electrodes and decrease the electrode width are taken in electrode design. These measures have changed the electric field distribution inside the sensor. When the sensor is put in regions with high electric field intensity, the insulating ability of sensor electrode structure should be studied [17,18,19]. Therefore, simulation calculation should be conducted for the electric field intensity between electrodes, and the simulation result is presented in Figure 12, where 1 represents the diameter of 10 kV electrified body; 2 refers to the air gap between electrode and sensor; and 3 indicates the spacing between sensor support and innermost electrode. According to the analysis, the electric field intensity between electrodes is far lower than the electric field intensity from electrode surface to the innermost side of sensor. In order to clearly reflect the electric field distribution between electrodes, the maximum ruler of the calculation result is 200 V/m, but the actual electric field intensity from electrode surface to the innermost side of sensor is far higher than 200 V/m. Table 2 shows the relative dielectric constant and critical field intensity of medium involved in the calculation. According to the distribution of electric field intensity, the maximum electric field intensity appears at the junction part of the outermost electrode and sensor support (50 V/m) as well as the interface of the sensor support and air (150 V/m). The electric field intensity between electrodes is quite low, almost equal to 0. Therefore, such structure can meet the insulation design between electrodes.

**Table 2:** Medium parameters.

Medium	Relative Permittivity	Critical Electric Field (kV/cm)
Epoxy (PCB)	3.6	200–300
Air	1	25–30

#### 4.2.2. Insulation Design of Sensor Support

**Figure 13:**
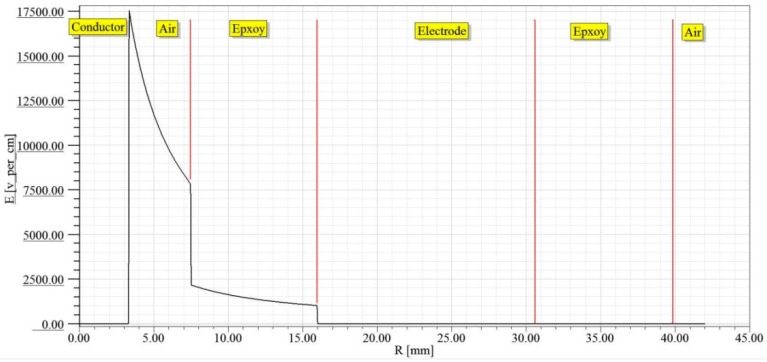
Insulation design simulation result of electrode.

**Figure 14:**
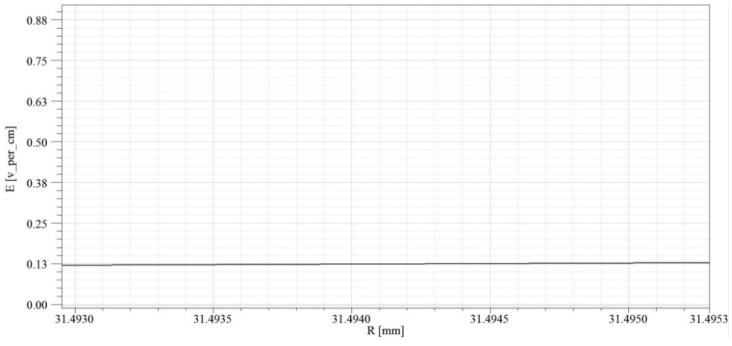
Internal electric field intensity distribution of sensor electrode.

Figure 13 shows the electric field intensity distribution of the electrified body under the voltage grade of 10 kV in electrified conductor (conductor), conductor and sensor air gap (air), innermost electrode and support of sensor (epxoy), sensor electrode (electrode), outermost electrode and support of sensor (epxoy), and sensor and air (air) successively. According to the simulation result, the maximum value of electric field intensity appears in the sensor support part closest to the electrified body (epoxy resin) and the electric field intensity is about 8000 V/m. As per Table 1, it is far lower than the critical field intensity of epoxy resin. Therefore, this structure can meet the insulation design of sensor support. The electric field intensity between electrodes inside the sensor is quite low, but it is not equal to 0. The electric field distribution after magnifying the coordinate system is presented in Figure 14.

### 4.3. Influence of Sensor Introduction on Measurement

When non-contact method is adopted to measure the voltage of electrified conductor, the use of sensor will inevitably influence electric field around the conductor and cause electric field distortion. Therefore, the distortion degree of electric field caused by the introduction of sensor should be studied [20,21,22].

**Figure 15:**
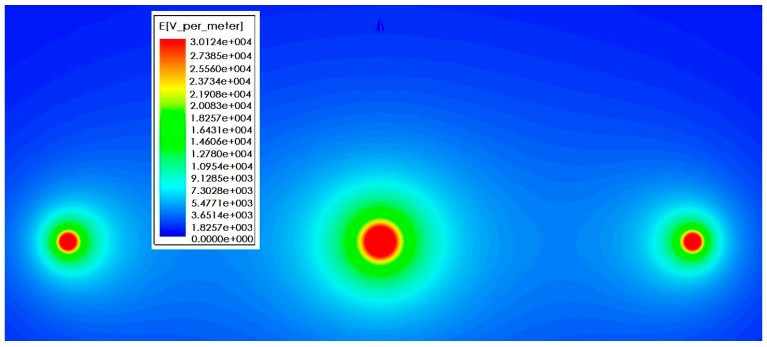
Electric field distribution without sensor.

**Figure 16:**
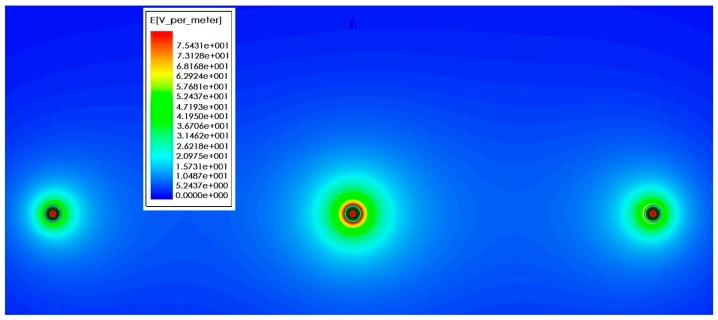
Electric field distribution after introducing sensor.

**Figure 17:**
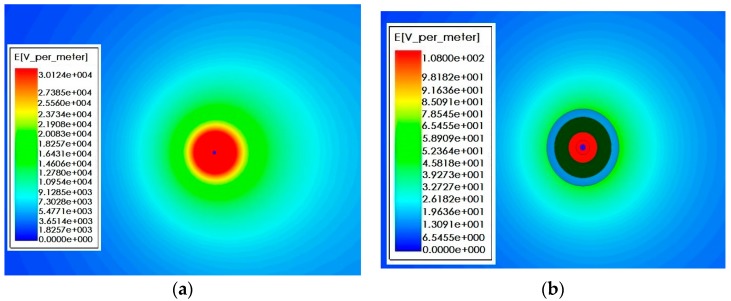
Influence of sensor on electric field. (**a**) A-phase electric field distribution without sensor; and (**b**) A-phase electric field distribution after introducing sensor.

Figure 15 shows the simulation result of electric field intensity in the cross section direction under horizontal arrangement of three-phase conducting wire. Sectional area of the wire is 35 mm², the wire spacing is 1.38 m, and the vertical height from the ground is 1.8 m. Its size and structure are consistent with the test platform actually established. During the simulation, the voltage frequency of three-phase conducting wire is 50 Hz and the voltage amplitude is 12 kV. In Figure 15, A-phase, B-phase and C-phase wires are presented from the left side successively, and the initial phases are 0°, −120° and −240°, respectively. It can be seen that when no sensor is added, electric field distribution around the three-phase conducting wire is uniform. Figure 16 shows electric field distribution around the three-phase conducting wire after sensor is installed on the line. According to the comparison, the introduction of sensor causes distortion to electric field distribution. The electric field intensity between phase and phase decreases obviously. As for the reason, the electric field line sent out by electric charges of the conductor ends in the electrode of voltage sensor. Figure 17a,b are diagrams for local electric field distribution of A-phase wire before and after sensor is used, respectively. The application of sensor changes the electric field distribution. However, according to Figure 17b, electric field distribution between the electrified conductor and voltage sensor is uniform, and no obvious electric field mutation is seen. In this section, the interphase electric field intensity is reduced by using the sensor, but no obvious electric field mutation is seen. The electric field distribution is still uniform.

Measurement points a and b are put 5 and 45 mm away from the left side of B-phase wire. Electric field intensity at the above two points is calculated at the same moment with the interval of 1 kV (the phase spacing of three phases is 120°) when the line voltage is within the range of 1–12 kV. Table 3 shows the electric field intensity when sensor is not used, and Table 4 presents the electric field intensity after sensor is used.

**Table 3:** Electric field intensity at A and B when sensor is not used.

Voltage Phase = 35 [deg] (kV)	Electric Field Intensity of Point A (kV/m)	Electric Field Intensity of Point B (kV/m)
1	2.10	0.15
2	4.20	0.30
3	6.30	0.48
4	8.39	0.60
5	10.49	0.75
6	12.59	0.90
7	14.69	1.04
8	16.79	1.19
9	18.89	1.34
10	20.99	1.49
11	23.089	1.64
12	25.189	1.79

**Table 4:** Electric field intensity at A and B when sensor is introduced.

Voltage Phase = 35 [deg] (kV)	Electric Field Intensity of Point A (kV/m)	Electric Field Intensity of Point B (kV/m)
1	13.35	0.008
2	25.86	0.012
3	39.00	0.016
4	52.00	0.019
5	65.00	0.023
6	78.00	0.028
7	90.99	0.032
8	103.99	0.035
9	116.99	0.040
10	129.98	0.045
11	132.15	0.058
12	155.99	0.062

**Figure 18:**
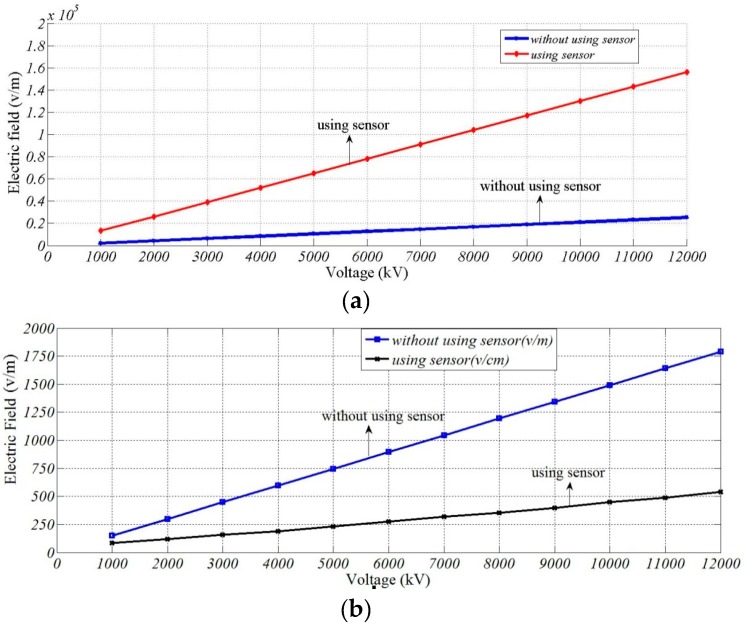
Fitting curve of electric field intensity and voltage grade: (**a**) phase A; and (**b**) phase B.

The linearity fitting curve graph shown in Figure 18 is drawn according to the results in Table 2 and Table 3.

Least-square fitting is conducted for electric field intensity data at A and B acquired before and after sensor is introduced, and Equations (18) and (19) are obtained, where x means electric field intensity of A and B before using the sensor, and y means electric field intensity of A and B after using the sensor. In the equation the constant term is generated under the combined action of boundary condition setting and fitting method when simulation model is established.

(18) y=6.0291x+1.06

(19) y=0.0317x+0.7024

According to the above simulation result, a linear relation is presented between electric field intensity around the circuit and line voltage when sensor is not used. When voltage sensor designed in this paper is used, a linear relation is still presented between electric field intensity around the circuit and line voltage. Therefore, though the use of voltage sensor will influence the electric field to be measured, electric field distribution is still uniform, and electric field intensity and line voltage present linear changes.

## 5. Experiment

The working performance of non-contact voltage sensor should be verified by experiment. The experiment is composed of sensor linearity test and sensor measurement accuracy test.

**Figure 19:**
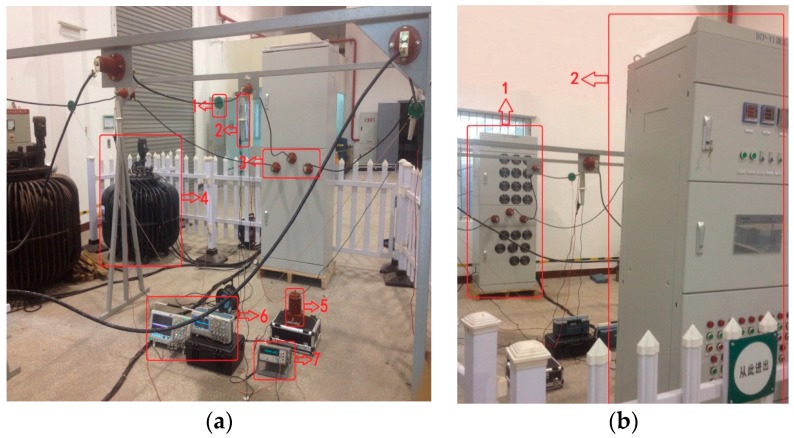
Experimental platform structure: (**a**) experimental platform structure 1; and (**b**) experimental platform structure 2.

The experimental platform is shown in Figure 19. In Figure 19a, 1 refers to the voltage sensor and this sensor is installed on all three-phase lines. Through design of the voltage sensor, its voltage division ratio is set as 1500:1. 2 represents the high-voltage probe of P6051A, its voltage division ratio is set as 1000:1. 3 indicates the wiring terminal of three-phase line. 4 denotes the transformer, its rated capacity is 50 kVA, and the output voltage of transformer can be adjusted through the control cabinet. 5 is the standard voltage transformer. 6 signifies the oscilloscope. 7 means the high-precision digital multimeter. In Figure 19b, 1 refers to the load part composed of resistive load and capacitive load, and the load part is equipped with a large number of fans for heat dissipation and 2 represents the control cabinet which can control the transformation ratio of transformer. Stepless adjustment can be realized for the voltage within the range of 0–35 kV. Meanwhile, the control cabinet can also switch the load, and the line current can change within the range of 0–200 A through the matching relation between voltage and load. Thus voltage and current of three-phase changes can coexist in the line and the purpose of simulating actual three-phase transmission line will be realized. Moreover, the control cabinet can realize real-time display for voltage, current and power factor of different phases. Sectional area of the wire is 35 mm², the distance from the wire to the ground is 1.8 m, and the spacing between phase and phase is 1.38 m.

### 5.1. Sensor Linearity Test

Sensor linearity test aims to study whether the detection value of sensor and the voltage of conductor to be measured still present linear changes when the use of sensor causes electric field distortion.

**Figure 20:**
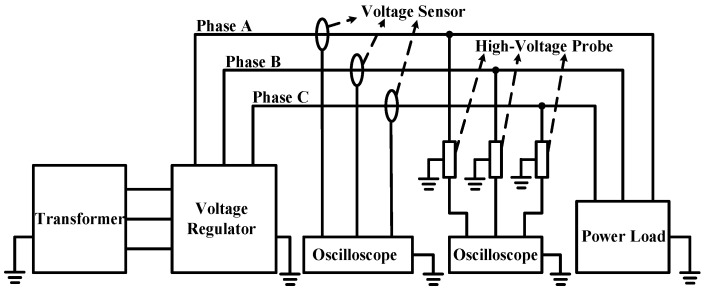
Schematic diagram for the structure of linearity test platform.

The schematic diagram for experimental platform is shown in Figure 20. The high-voltage probe is connected to A-phase, B-phase and C-phase wires, respectively; meanwhile, lines of the three phases all pass through the non-contact voltage sensor. Output connection to oscilloscope is realized between the high-voltage probe and sensor, the voltage of control cabinet is adjusted, and waveform and phase difference data are recorded every 1 kV within the range of 1–12 kV.

**Figure 21:**
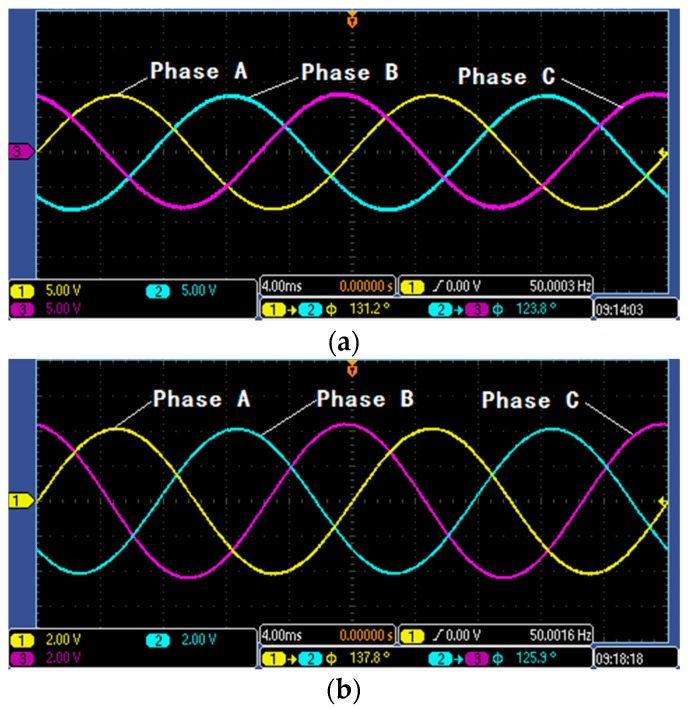
Waveform figure: (**a**) Signal of high-voltage probe under 8 kV; and (**b**) signal of voltage sensor under 8 kV.

Figure 21a shows the line voltage waveform detected by high-voltage probe under the voltage grade of 8 kV, and Figure 21b shows the detection signal waveform of voltage sensor under the voltage grade of 8 kV. According to Figure 21, detection signal of the designed voltage transformer has the same waveform with the actual line voltage signal detected by high-voltage probe, and the voltage division ratio of voltage transformer is 1500:1.

Besides resistive load, capacitive and inductive loads also exist in the part of experimental platform design and platform load implementation, so there are some errors between phase and phase in the three lines. Therefore, the difference is not exactly 120°.

**Figure 22:**
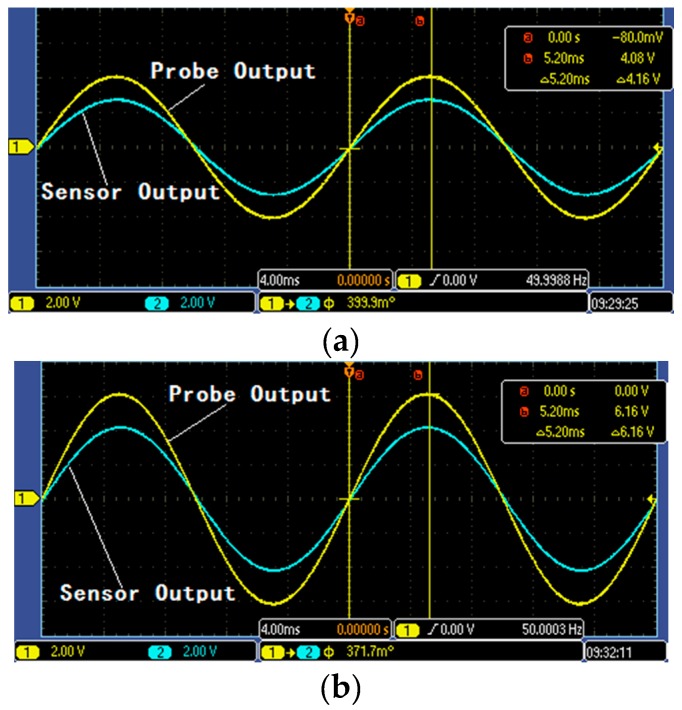
Comparison figure of high-voltage probe and voltage sensor: (**a**) signals comparison under 4 kV; and (**b**) signals comparison under 6 kV.

Figure 22a shows the output waveform of A-phase line high-voltage probe and non-contact voltage sensor under the voltage grade of 4 kV, and Figure 22b shows the output waveform of A-phase line high-voltage probe and non-contact voltage sensor under the voltage grade of 6 kV.

The phase error of the three-phase line between these two under different voltage grades is gained, as shown in Table 5. In the table, *φ_S_* indicates the output voltage phase of voltage sensor and *φ_H_* signifies the output voltage phase of high-voltage probe. According to Table 5, voltage sensor works under self-integration mode. According to Table 4, the maximum phase error of voltage sensor and high-voltage probe is 33.9′ and the average error is 27.925′. The phase detection accuracy of voltage sensor under steady state meets the accuracy class of 0.5-level voltage sensors.

**Table 5:** Phase error.

Voltage (kV)	*φ_H_*-*φ_S_* (')
1	33.2
2	30.7
3	25.4
4	23.9
5	25.7
6	22.3
7	31.7
8	32.6
9	25.4
10	22.8
11	33.9
12	27.5

### 5.2. Sensor Measurement Accuracy Test

Sensor measurement accuracy test aims to verify the measurement accuracy grade of voltage sensor, and it is the important index for the working performance of voltage sensor.

**Figure 23:**
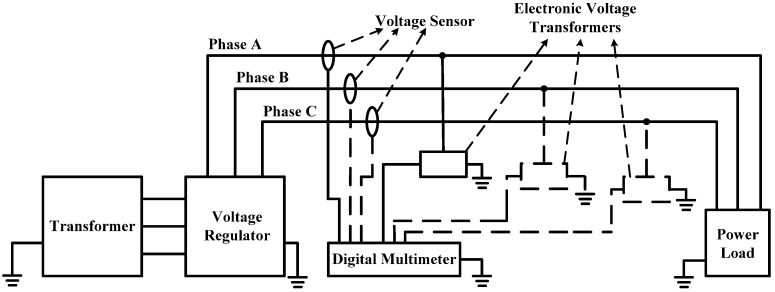
Schematic diagram for the structure of accuracy test platform.

The experimental platform of simulating three-phase transmission line shown in Figure 19 is still adopted, but the verification test is conducted by adopting the method of phase measurement. Figure 23 is the schematic diagram for the structure of sensor measurement accuracy test platform. The standard voltage transformer adopts EVT1-20 electronic voltage transformer. The rated voltage of primary side is 20 kV, the working frequency is 50 Hz, the rated phase shift is 0, the rated output of secondary side is 0–4 V(AC), and the accuracy class is 0.2. 34410A high-performance digital multimeter of Keysight Truevolt series is adopted as high-precision digital multimeter, and the measurement accuracy of measuring the alternating voltage of 50 Hz and 0–50 V is 0.006%. Therefore, the above experimental equipment can guarantee the accuracy of the test conclusion.

The output ends of standard electronic voltage transformer and non-contact voltage sensor are connected to digital multimeter, the three-phase voltage is adjusted, and the output results of A-phase, B-phase and C-phase standard voltage transformer as well as the output results of non-contact voltage sensor designed in this paper are recorded every 1 kV within the range of 1–12 kV. The ratio error *ε*% between sensor signal and actual voltage signal is gained according to the provisions in IEC60044-7 standard, and its definition is as follows:

(20) $$\varepsilon \text{\%=}\frac{KU_{S}-MU_{H}}{MU_{H}}\times 100\%$$

where *K* refers to the voltage division ratio of voltage sensor designed in this paper and the rated transformation ratio after calibration is 1500:1; *Us* means the detection signal of voltage sensor; *M* signifies the standard electronic voltage transformer and the transformation ratio is 5000:1; and *U_H_* denotes the output voltage of standard electronic voltage transformer. The experimental results are shown in Table 6, Table 7 and Table 8.

**Table 6:** Results of Phase A.

Voltage (kV)	*Us* (kV)	*U_H_* (kV)	*ε*%
1	0.6582	0.1967	0.37
2	1.3245	0.3961	0.31
3	1.9763	0.5912	0.28
4	2.6496	0.7929	0.24
5	3.4023	1.0231	0.21
6	4.0518	1.2138	0.14
7	4.6164	1.3834	−0.11
8	5.3271	1.6010	−0.17
9	6.0846	1.8301	−0.21
10	6.6743	2.0079	−0.23
11	7.3257	2.2027	−0.27
12	8.0710	2.4293	−0.33

**Table 7:** Results of Phase B.

Voltage (kV)	*Us* (kV)	*U_H_* (kV)	*ε*%
1	0.6732	0.2011	0.41
2	1.3411	0.4008	0.37
3	2.0177	0.6030	0.39
4	2.6714	0.7994	0.25
5	3.3376	0.9987	0.26
6	4.0365	1.2088	0.18
7	4.6585	1.3943	0.23
8	5.3436	1.6014	0.17
9	6.0094	1.7990	0.21
10	6.6832	2.0106	−0.28
11	7.3431	2.2080	−0.23
12	7.9763	2.4018	−0.37

**Table 8:** Results of Phase C.

Voltage (kV)	*Us* (kV)	*U_H_* (kV)	*ε*%
1	0.6641	0.1998	−0.31
2	1.3276	0.3994	−0.27
3	1.9883	0.5985	−0.33
4	2.6475	0.7958	−0.21
5	3.3416	1.0062	−0.31
6	4.0117	1.2010	0.21
7	4.6774	1.3992	0.27
8	5.3169	1.5901	0.31
9	6.0712	1.8154	0.33
10	6.6573	1.9918	0.27
11	7.3448	2.1955	0.36
12	7.9659	2.3829	0.29

According to the above two tests, some errors exist in detection signals of the non-contact voltage sensor designed in this paper and standard electronic voltage transformer, and the errors mainly come from two sources. On the one hand, though the sensor is specially designed, the position of its electrode arrangement has inevitable errors due to the restriction of craftsmanship. As a result, errors between transformer phase position and actual voltage phase are caused. However, such errors can be ignored in actual use. On the other hand, complete coaxial arrangement cannot be realized between the voltage sensor and line, so the electric field lines passing through sensor electrode are imbalanced and the measurement accuracy will be affected. However, such errors can be ignored in actual use. The voltage ratio error of the non-contact voltage sensor designed in this paper within the voltage range of 0–12 kV is *ε*% < 0.5%, and the phase difference is *φ* < 35′. Therefore, the measurement accuracy of such non-contact voltage sensor can meet the actual measurement requirement during steady state measurement.

## 6. Conclusions and Prospect

According to this paper, the following conclusions can be gained: 

(1) When non-contact voltage sensor is applied to steady state voltage measurement within 12 kV, the phase error is smaller than 35′, which meets the phase detection accuracy class of 0.5-level voltage transformer used for measurement.

(2) When non-contact voltage sensor is applied to steady state voltage measurement within 12 kV, the ratio error *ε*% is <0.5%, so the accuracy of steady state measurement meets the accuracy class of 0.5-level voltage transformer used for measurement.

(3) Through simulation and experiment, structure design of sensor is optimized, voltage division ratio of voltage sensor is increased, insulation design difficulty of sensor is reduced, and the sensor is able to work under self-integration mode.

In the future, in-depth studies will be made into the aspects of standardization, anti-interference and signal processing circuit of non-contact voltage sensor. In order to meet the electrified installation requirement of sensor, open-type design should be conducted for the sensor electrode; in order to solve the coaxial arrangement problem of sensor and line, a corresponding fixture should be designed. 
